# Bright squeezed light in the kilohertz frequency band

**DOI:** 10.1038/s41377-025-02013-7

**Published:** 2025-09-08

**Authors:** Ruixin Li, Bingnan An, Nanjing Jiao, Junyang Liu, Lirong Chen, Yajun Wang, Yaohui Zheng

**Affiliations:** 1 State Key Laboratory of Quantum Optics Technologies and Devices, Institute of Opto-Electronics, Shanxi University, 030006 Taiyuan, China; 2 Collaborative Innovation Center of Extreme Optics, Shanxi University, 030006 Taiyuan, Shanxi China

**Keywords:** Quantum optics, Photonic devices

## Abstract

The dominant technical noise of a free-running laser practically limits bright squeezed light generation, particularly within the MHz band. To overcome this, we develop a comprehensive theoretical model for nonclassical power stabilization, and propose a novel bright squeezed light generation scheme incorporating hybrid power noise suppression. Our approach integrates broadband passive power stabilization with nonclassical active stabilization, extending the feedback bandwidth to MHz frequencies. This hybrid technique achieves an additional 9 dB technical noise suppression, establishing critical prerequisites for broadband bright squeezed light generation. Finally, a -5.5 dB bright squeezed light at 1 mW with kHz-MHz squeezing bandwidth was generated. The experimental results show excellent agreement with theoretical predictions, which represent we have comprehensively demonstrated a milliwatt-order bright squeezed light across kHz-MHz frequencies. Our work enables new quantum metrology applications and paves the way for next-generation quantum-enhanced technologies.

**Figure:**
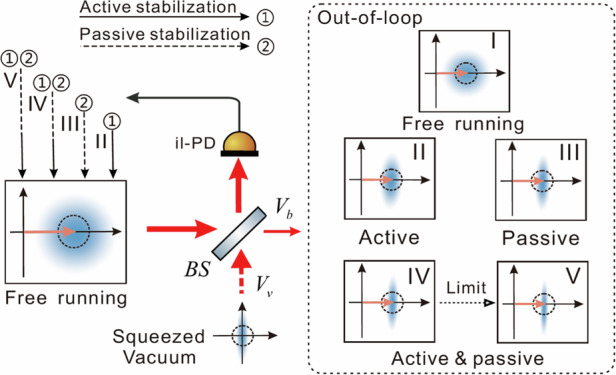
Nonclassical hybrid passive–active power stabilization enables milliwatt-level bright squeezing across kHz–MHz band

Nonclassical hybrid passive–active power stabilization enables milliwatt-level bright squeezing across kHz–MHz band

## Introduction

Squeezed states of light describe a unique quantum state in which the uncertainty in one quadrature (amplitude or phase) is reduced below the shot noise limit (SNL), while its conjugate quadrature is amplified^1,2^. By exploiting their sub-shot-noise property, squeezed states of light have become an extremely valuable resource for various applications in quantum information technologies^3–10^. In the first scenario, the laser power is constrained to relatively low levels due to the damage thresholds of samples under test, such as biological imaging^11–14^ and medical diagnostic^15,16^; nonlinear effects in optical fiber impacting fiber communications^17–19^ and clock synchronization^20,21^; and payload limitations on satellite and airborne platform^22^. In the second scenario, higher laser power inevitably introduces excess technical noise that degrades—rather than improves system sensitivity, as seen in plasmonic sensing^23–26^. In the third scenario, current technological limitations prevent arbitrary increases in laser power, e.g., laser Doppler anemometry^27^. However, beyond noise suppression^28–30^, the frequency band and output power of the squeezed light are critical for specific applications, such as biological tracking among kHz to MHz band^31–34^, microcantilever displacement measurement up to hundreds of kHz band^35,36^ and optomechanical coupling in the kHz frequency range^37,38^. Moreover, all these applications require laser output power in the microwatt-to-milliwatt range.

To date, the maximum squeezing strength is −15 dB at the MHz sideband frequencies of 1064 nm wavelength^30^. Beyond the squeezing strength, extending the bandwidth to lower frequencies has gained significant interest, particularly in quantum precision measurement^39^ and sensing^40,41^. Current approaches for generating sub-MHz squeezed light exploit a key mechanism of immunity to nonlinear pump-seed noise coupling^42–44^, while blocking the transfer of the seed laser’s low-frequency technical noise^45^. This requires replacing the seed beam at the carrier frequency with a vacuum field (no seed input) in optical parametric oscillators (OPOs). Consequently, the OPO configurations are inherently limited to producing squeezed vacuum states^43,44^. Critically, these squeezed vacuum states exhibit extremely low power, which is insufficient for biological sensing and optomechanical applications requiring microwatt-to-milliwatt power levels^31–38^.

Bright squeezed light, exhibiting sub-shot-noise quantum noise (like squeezed vacuum) combined with significant optical power, is essential for enhanced sensitivity. Experimentally, a narrow-band bright squeezed state (−0.7 dB squeezing) was generated using radiation-pressure-driven interactions between coherent light and a mechanical oscillator^46^. Conventionally, bright squeezed light is prepared via passive interference of squeezed vacuum with a laser beam on a beam splitter (BS)^13,38^. This method demands a quantum-noise-limited laser beam; otherwise, inherent low-frequency classical noise degrades the squeezing level. Even with an ideal laser, quantum noise persists due to vacuum noise injection from the empty port of the beamsplitter and shot noise from the output beam. Crucially, BS-introduced vacuum noise caps the maximum achievable squeezing. To date, only −2.6 dB of bright squeezed light at 25 μW has been demonstrated in the 2–200 kHz range^38^. Theoretically, infinite-gain active feedback could fully compensate laser technical noise, extending squeezing bandwidth to lower frequencies, while limiting noise to in-loop electronic noise and out-of-loop quantum noise penalty^47,48^. A trade-off exists between the lower frequency limit and out-of-loop power, e.g., tunable via BS ratio in the feedback loop. High feedback gain is a prerequisite for increasing out-of-loop power. Based on a pioneering theory^47^, nonclassical power stabilization, extracting the error signal via a 50:50 BS, achieved −5.7 dB squeezed light at 9.9 mW within 6–20 kHz band^49^. However, an inherent gain-bandwidth contradiction in control loops^50,51^ restricts squeezing bandwidth to ∼tens of kHz.

This article presents a comprehensive theoretical model for bright squeezed light generation and introduces a novel framework for preparing bright squeezed light across the kHz–MHz frequency band. Our approach employs a hybrid passive-active nonclassical stabilization scheme that synergistically combines the high loop gain of active stabilization with the broadband noise suppression of a passive technique. The hybrid scheme suppresses laser technical noise from −125 to −166 dB/Hz in the kHz band, which is 10 dB below the relative shot noise limit of −156 dB/Hz. The ultra-low intrinsic noise relaxes the control loop gain requirements, enabling MHz-range loop bandwidth. Implementing nonclassical stabilization through a 99:1 beamsplitter, we demonstrate a −5.5 dB bright amplitude squeezed light at 1 mW optical power and kHz to MHz bandwidth. Experimental results show excellent agreement with theoretical predictions. We have comprehensively demonstrated a milliwatt squeezed light across such broadband frequencies. This breakthrough enables new quantum metrology applications and paves the way for novel quantum-enhanced technologies.

## Results

### Principle analysis

**Fig. 1:**
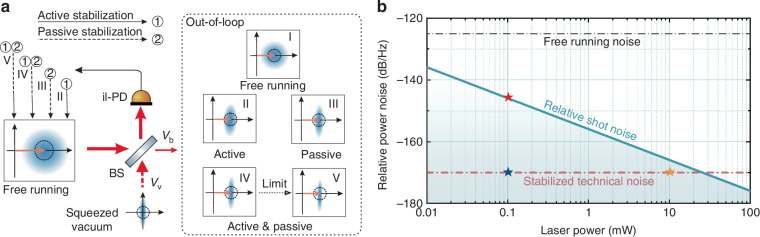
Strategy for generating bright squeezed light and the technical noise comparison with SNL. **a** Simplified schematic of squeezed light generation integrating multiple noise suppression techniques. (I) free-running noise; (II) active stabilization at detectable power; (III) passive stabilization; (IV) active and passive stabilization at detectable power of 10 mW, limited by the saturation power of the detector; (V) active and passive stabilization at the detectable power of 99 mW, assuming no saturation power limit; il-PD in-loop photodetector; BS beam splitter. **b** Relationship between the laser power and relative shot noise. Red star: quantum noise penalty of the out-of-loop beam; Blue star: technical noise after active feedback; Yellow star: in-loop electronic noise

The schematic diagram of bright squeezed light generation is shown in Fig. 1a, which depicts a nonclassical active feedback scheme with the assistance of a squeezed vacuum. Prior to the active feedback stage, the free-running laser is pre-stabilized by employing a passive noise stabilization technology. One of its output beams serves as the in-loop sensing beam, which is detected by the in-loop photodetector (il-PD) to generate the error signal for active stabilization. This error signal is used to suppress the amplitude noise below the shot noise of the out-of-loop beam, thereby enabling the extraction of sub-shot-noise light at the out-of-loop port. In this process, the squeezed vacuum not only suppresses the vacuum in the in-loop beam but also provides enhanced quantum noise reduction for the out-of-loop beam compared with the passive interference scheme. Two critical noise sources determine the achievable squeezing strength: (i) the technical noise of the free-running laser in the kHz frequency band, and (ii) the quantum noise arising from vacuum fluctuations entering through the dark port of the BS, as well as the shot noise of the light transmitted from the BS. Based on the theoretical derivation of nonclassical feedback (see Supplementary Note 2), the amplitude quadrature variance of the light transmitted from the BS can be approximately expressed as^47^

1 $${V}_{b}({P}_{OOL})=\frac{{V}_{\nu }}{r}+\frac{{TN}_{OOL}}{{RSN}_{OOL}}$$

where ${P}_{{\rm{O}}{\rm{O}}{\rm{L}}}$, ${{TN}}_{{OOL}}$ and ${{\rm{R}}{\rm{S}}{\rm{N}}}_{{\rm{O}}{\rm{O}}{\rm{L}}}$ denote the power, relative technical noise (TN), and relative shot noise (RSN) of the out-of-loop (bright squeezed) beam, respectively. The relative shot noise is given by ${\rm{R}}{\rm{S}}{\rm{N}}=2h\nu /P$, where $\nu$ is the laser frequency and $P$ is the total input power, as shown in Fig. 1b. ${V}_{\nu }$ is the noise variance of the squeezed vacuum, and ${r}$ denotes the power reflectivity of the BS. The first term of Eq. (1) represents the lower bound of the noise variance, which depends on both the squeezing strength of the quantum state and the BS splitting ratio. The second term originates from the technical noise of the laser, which must be suppressed below the SNL of the out-of-loop beam.

In our scheme, the laser’s technical noise is passively pre-stabilized near the SNL in the kHz to MHz band by exploiting a second harmonic generator (SHG). This not only relaxes the requirements for a high-gain broadband feedback control loop, but also extends the squeezing bandwidth into the MHz range. The noise suppression mechanism is based on a nonlinear three-step-photon-recycling (TSPR) process (see details in the “Materials and methods” section), which produces more than 100 mW of laser power for bright squeezed light generation^52^. After passive stabilization, the technical noise of the laser can be approximately expressed as

2 $${TN}_{OOL-P}=g(f)\cdot {TN}_{F}$$

where $g(f)$ is the noise reduction factor of TSPR, and ${TN}_{F}$ is the technical noise of free-running laser (see Supplementary Note 1). The factor $g(f)$ is approximately frequency-independent across the kHz band^52^. Subsequently, integrating with nonclassical active feedback ($G\left(f\right)\gg 1$), the technical noise of the out-of-loop laser beam can be inferred as (see Supplementary Note 2)

3 $$T{N}_{OOL-P\& A}=g(f)\cdot T{N}_{F}\cdot \frac{1}{{|1-\sqrt{r}\cdot G(f)|}^{2}}+RS{N}_{IL}{V}_{{\rm{e}}}$$

where ${{\rm{R}}{\rm{S}}{\rm{N}}}_{{\rm{I}}{\rm{L}}}$ and ${V}_{{\rm{e}}}$ are the RSN relating to the detected laser power and electronic noise of the il-PD, respectively. $G(f)$ is the frequency-dependent feedback gain of the active control loop, which is limited by the inherent time delay of the system, resulting in a finite feedback bandwidth ${f}_{{\rm{B}}}$. As a rule of thumb, its inherent mechanism restricts the maximum achievable noise suppression at Fourier frequency $f$ to a factor of approximately ${f}_{{\rm{B}}}/f$ ^50,51^. The first term in Eq. (3) denotes the residual technical noise of the out-of-loop unit, which in principle can be fully eliminated by an infinitely large feedback gain *G*(*f*). The second term corresponds to the electronic noise of the feedback control loop, primarily arising from the photodetector electronic noise imprinted onto the out-of-loop beam. With infinite feedback gain, the out-of-loop technical noise ${{\rm{T}}{\rm{N}}}_{{\rm{O}}{\rm{O}}{\rm{L}}}$ can be suppressed below the SNL of the out-of-loop beam. In this regime, the electronic noise of the photodetector (${{\rm{R}}{\rm{S}}{\rm{N}}}_{{\rm{I}}{\rm{L}}}{V}_{{\rm{e}}}$) becomes the dominant limiting factor for the squeezing strength, particularly at higher power output. It may lead the ${{\rm{R}}{\rm{S}}{\rm{N}}}_{{\rm{O}}{\rm{O}}{\rm{L}}}$ to approach the electronic noise, and no squeezing can be observed. It follows the fact that the electronic noise associated with the il-PD is imprinted onto the beam transmitted from the BS, which makes the electronic noise become the dominant limit for generating bright squeezed light.

Interestingly, if the condition of $r > {V}_{\nu }$ and ${{\rm{T}}{\rm{N}}}_{{\rm{O}}{\rm{O}}{\rm{L}}} < < {{\rm{R}}{\rm{S}}{\rm{N}}}_{{\rm{O}}{\rm{O}}{\rm{L}}}$ is satisfied, the scheme combined with a squeezed vacuum can be used to produce a sub-shot noise light, effectively transforming the squeezed vacuum into amplitude-squeezed light. The key point is to suppress the$\,{{\rm{T}}{\rm{N}}}_{{\rm{O}}{\rm{O}}{\rm{L}}}$ far below ${{\rm{R}}{\rm{S}}{\rm{N}}}_{{\rm{O}}{\rm{O}}{\rm{L}}}$. As an example, we consider the experimental parameters in our experimental setup, where the BS’s power reflectivity $r$ is 99%. A passively stabilized laser beam with a power of 10 mW is divided into two beams by the BS. The 9.9 mW one serves as the in-loop sensing beam detected by the il-PD, whose electronic noise is marked by the yellow star in Fig. 1b. The optical power of the out-of-loop laser is 0.1 mW, and its quantum noise penalty (${V}_{\nu }/r$) is indicated by the red star in Fig. 1b. Under infinite feedback gain, ${{\rm{T}}{\rm{N}}}_{{\rm{O}}{\rm{O}}{\rm{L}}}$ can be completely suppressed to the electronic noise level of the il-PD, shown as the blue star in Fig. 1b, which lies 24 dB lower than ${{\rm{R}}{\rm{S}}{\rm{N}}}_{{\rm{O}}{\rm{O}}{\rm{L}}}$. Consequently, the influence of the second term of Eq. (1) on squeezing strength can be neglected. Therefore, it establishes the physical conditions for bright squeezed light generation.

**Fig. 2:**
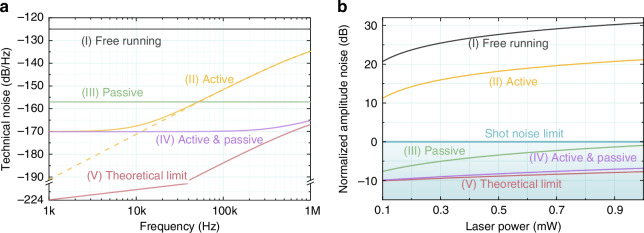
Analysis results of the technical and squeezed noises under different conditions. **a** Dependence of the out-of-loop technical noise ${{\rm{T}}{\rm{N}}}_{{\rm{O}}{\rm{O}}{\rm{L}}}$ on the analysis frequency. Experimental parameters are listed as: BS splitting ratio: 99:1; feedback bandwidth: 2 MHz; free-running noise: −125 dB/Hz; detectable in-loop power: 10 mW; photodetector electronic noise is −170 dB/Hz; squeezing strength: −10.5 dB. The final theoretical limit is simulated by considering a 99 mW optical power detected by the il-PD without electronic noise (trace V). In practice, the detectable power is restricted to the 10 mW saturation power of the il-PD with non-negligible electronic noise (traces I–IV). The dashed line is calculated by the factor of ${f}_{{\rm{B}}}/f$. **b** Dependence of the squeezing strength of bright squeezed light at analysis frequency of 1 MHz on the output power, which represents the maximum measurement frequency in the squeezing bandwidth. All these results are inferred from **a** and Eqs. (1)–(3), and are normalized to SNL

To further clarify the mechanism, we simulate the technical and squeezed noise for different stabilization schemes based on Eqs. (1)–(3), with the experimental parameters listed in the caption of Fig. 2. Figure 2a presents the theoretical results of ${{\rm{T}}{\rm{N}}}_{{\rm{O}}{\rm{O}}{\rm{L}}}$. Trace (I) is the technical noise of the free-running laser at the il-PD’s saturation power of 10 mW, corresponding to an electronic noise level of −170 dB/Hz. Firstly, exploiting single passive noise stabilization^52^, the technical noise is suppressed to −157 dB/Hz (trace (III)) across the kHz–MHz frequency band, yielding a noise reduction of 32 dB. However, the gap between the technical noise and electronic noise is only 13 dB, indicating that ${{\rm{T}}{\rm{N}}}_{{\rm{O}}{\rm{O}}{\rm{L}}}$ remains the main limitation for bright squeezing generation in the passive interference regime. Moreover, as the optical power increases, the squeezing strength decreases rapidly, as shown in trace (III) of Fig. 2b. Secondly, the technical noise, with dependent nonclassical active noise stabilization, is calculated as trace (II). The feedback bandwidth of our control loop is *f*_B_ = 2 MHz. The best noise performance for different $f$ with optimized feedback loop is shown as the dashed line in trace (II) of Fig. 2a, where the feedback gain decreases linearly with the increase of Fourier frequency $f$, and thus the technical noise suppression level also decreases. In real applications, the active feedback noise level is further limited by the electronic noise of the photodetector, which constrains the technical noise to the electronic noise floor for finite feedback gain below 20 kHz, shown as the solid line in trace (II) of Fig. 2a. As $f$ is increased to 50 kHz, the technical noise rises to the shot noise level of the out-of-loop beam, beyond which the squeezing is rapidly submerged by the technical noise floor, as seen in trace (II) of Fig. 2b. Therefore, the squeezing bandwidth is always limited to within 100 kHz by using pure active feedback control regime^38,49^. Finally, by integrating passive stabilization into the nonclassical active scheme, the noise suppression bandwidth can be extended to the MHz range (trace (IV) in Fig. 2a). Under this operation, a broadband, high-power bright squeezed light can be generated within the MHz band (trace (IV) in Fig. 2a, b). Assuming no electronic noise limitation and using a passively pre-stabilized laser beam with an available power of 99 mW detected by the il-PD, ${{\rm{T}}{\rm{N}}}_{{\rm{O}}{\rm{O}}{\rm{L}}}$ can be suppressed to −224 dB/Hz at 1 kHz, as shown in trace (V) of Fig. 2a.

According to Eqs. (1)–(3) and the results in Fig. 2a, we simulated the dependence of the squeezing strength on the output power at a Fourier frequency of 1 MHz (Fig. 2b), which represents the worst-case of noise reduction across the kHz–MHz band. The results indicate that an individual noise stabilization regime is insufficient for generating broadband bright squeezed light with MHz bandwidth and milliwatt-level output power. In contrast, with the nonclassical hybrid power stabilization scheme, it is feasible to achieve −6.5 dB bright squeezed light across the kHz–MHz band at 1 mW output power. By comparing traces (IV) with theoretical limit traces (V) in Fig. 2a and b, it can be seen that higher squeezing strength and output power can be attained simultaneously by increasing the saturation power and reducing the electronic noise of the il-PD. This new perspective, based on the theoretical models, enables a comprehensive and efficient optimization of the practical experimental parameters and conditions for nonclassical power stabilization. With this comprehensive model, the final noise performance of the out-of-loop beam is co-determined by the residual technical noise of the optical field, the electronic noise of the il-PD, and the quantum noise penalty of the out-of-loop.

### Experimental setup

**Fig. 3:**
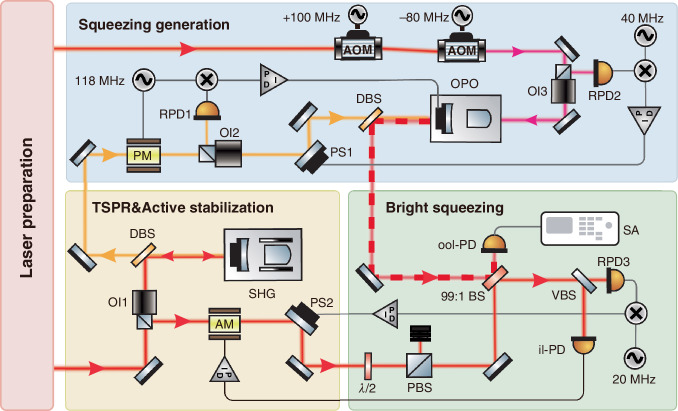
Experimental setup of bright squeezed light generation. PM phase modulator, AM amplitude modulator, AOM acousto-optic modulator, OI optical isolator, RPD resonant photodetector, PID proportional–integral–derivative, SHG second harmonic generator, *λ*/2 half-wave plate, PBS polarization beam splitter, DBS dichroic beam splitter, VBS variable beam splitter, OPO optical parametric oscillator, PS phase shifter, il-PD in-loop photodetector, ool-PD out-of-loop photodetector, 99:1 BS 99:1 beam splitter, SA spectrum analyzer

Figure 3 illustrates the schematic diagram of bright squeezed light generation. It mainly includes three main units: (1) a hybrid technical noise suppression scheme, consists of a passive stabilization technology based on a TSPR technology with a SHG, and a nonclassical active stabilization technology; (2) a squeezed vacuum generator includes an OPO for squeezed vacuum state preparation and a coherent control loop for relative phase locking; (3) a bright squeezed state generation and characterization unit. The single-frequency laser source for squeezed light preparation is a 1550 nm continuous-wave (CW) fiber laser (NKT, Koheras BASIK X15) with 1 W output power. Prior to downstream applications, the laser transmits into a mode cleaner (MC) in the laser preparation stage to improve the purity of the spatial fundamental mode and polarization, as well as to filter amplitude and phase noises above the MC linewidth^53^. Subsequently, approximately 500 mW of the laser beam is injected into the SHG with a conversion efficiency of 70%. The generated 775 nm second harmonic wave serves as the pump source for the OPO to generate a squeezed vacuum state. The residual 1550 nm laser reflected from SHG acts as the passively stabilized laser beam, with a power of about 110 mW, and is isolated by optical isolator (OI1)^52^. This beam then passes through an amplitude modulator (AM) actuator and is coupled onto a 99:1 beam splitter (99:1 BS) with the squeezed vacuum. The reflected interference field serves as the sensing beam of the in-loop unit, while the transmission beam is utilized for out-of-loop applications or amplitude noise characterization. A half-wave plate (*λ*/2) and a polarization beam splitter (PBS) are positioned between the AM and 99:1 BS to enable continuous adjustment of the incident laser power at the 99:1 BS. Before reaching the il-PD, the beam passes through a variable beam splitter (VBS) for power attenuation. The VBS output is injected into the il-PD, whose photocurrent is fed back to the AM to perform nonclassical active noise stabilization. Both the in-loop and out-of-loop photodetectors are commercial detectors (Newport model 2053) with a saturation power of 10 mW, electronic noise of −170 dB/Hz, and a response frequency range from 1 kHz to 3 MHz. In our experiment, the photodiodes inside the two photodetectors are replaced with high-quantum-efficiency ones custom-made by Laser Components GmbH in Germany (detailed parameters can be found in “Materials and methods”). A proportion–integration–differentiation (PID) controller (Vescent model D2-125) with 2 MHz bandwidth is employed to provide high-gain feedback for amplitude noise suppression.

A squeezed vacuum state is prepared by a sub-threshold degenerate OPO via the phase-sensitive parametric down-conversion process. Its squeezing strength reaches −10.5 dB at pump power of 16 mW, by budgeting the losses and phase fluctuations in the system. The OPO contains a periodically poled potassium titanyl phosphate (PPKTP) crystal and a planoconcave mirror. Its threshold power is 20 mW, and it operates in double resonance at both 1550 and 775 nm wavelengths (further details can be found in refs. ^54–56^. The 775 nm laser is modulated by a phase modulator (PM) to generate a pair of ±118 MHz phase sidebands, which are injected into the OPO to serve as both the pump source and sensing beam for cavity length locking via the Pound–Drever–Hall (PDH) technology. A coherent control method is employed to stabilize the squeezing angle, thereby producing a stable amplitude squeezed state^57,58^. A 20 MHz frequency-shifted 1550 nm laser beam with 1 mW power acts as the phase sensing beam, which is generated by two acousto-optic modulators (AOMs) driven by a +100 MHz and a −80 MHz sinusoidal signals, respectively. The error signal is demodulated at 40 MHz and fed back to the phase shifter (PS1) to lock the relative phase between the pump and squeezed fields to *π*. The relative phase between the squeezed vacuum and the in-loop sensing beam is locked to zero using the same technique. Here, the pump power of the OPO is reduced to half of its threshold power, if not the larger anti-squeezing noise will lead to instable in the coherent control loop. Consequently, only −8.6 dB squeezed vacuum is utilized in the nonclassical hybrid stabilization regime (see Supplementary Note 4). Finally, a bright amplitude squeezed light is continuously generated at the transmission port of the 99:1 BS, which serves as the out-of-loop beam in our scheme.

### Experimental results

**Fig. 4:**
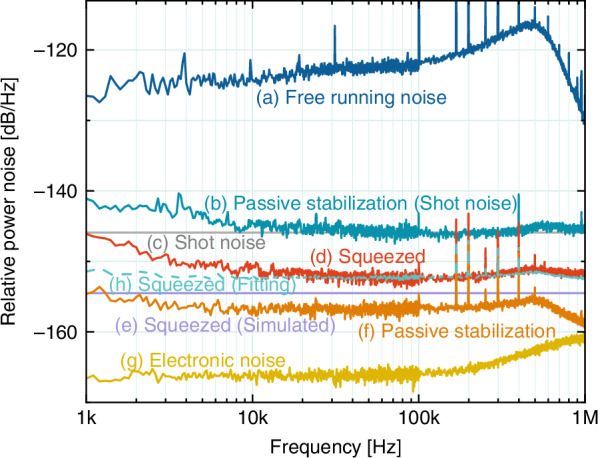
Measurement results of bright squeezed light at 100 μW with the passive interference method (pre-stabilized by SHG). Trace (a): measured technical noise of the free-running laser. Trace (b): measured RSN of the 100 μW optical power detected by ool-PD. Trace (c): Theoretical shot noise of 100 μW optical power. Trace (d): measured noise variance of the bright squeezed light across 2 kHz–1 MHz band. Trace (e): simulated noise variance of the bright squeezed light. Trace (f): technical noise of the laser passively stabilized by SHG. Trace (g): electronic noise of the ool-PD. Trace (h): uncorrelated sum of the noise sources (e–g). All measurements were performed using a spectrum analyzer (R&S FSW) at two frequency bands, 1–100 kHz and 100 kHz–1 MHz, with a resolution bandwidth (RBW) of 100 Hz and a video bandwidth (VBW) of 10 Hz

First of all, we coherently combine a passively stabilized 10 mW laser beam with a −10.5 dB squeezed vacuum at a 99:1 BS. As a result, we generate a −6.5 dB bright squeezed light with an output power of 100 μW and bandwidth of 1 kHz–1 MHz, as shown in Fig. 4. Trace (a) depicts the technical noise of the free-running laser at 100 μW. Trace (b) shows the shot noise level across the kHz band measured by the out-of-loop photodetector (ool-PD) after passive noise stabilization, compared with the −146 dB/Hz SNL of a 100 μW laser (trace (c)). Due to minor cavity detuning of the SHG, the noise spectrum below 10 kHz is slightly above SNL. The residual technical noise after passive stabilization (trace (f)) is −157 dB/Hz at detected power of 9.9 mW, which is 11 dB below its SNL (trace (c)). The electronic noise of the ool-PD (trace (g)) is −167 dB/Hz, ~20 dB below the SNL. The total technical noise in our scheme is the sum of the residual optical technical noise and photodetector’s electronic noise. These results clearly demonstrate that passive noise stabilization can be employed to generate bright squeezed light across the kHz–MHz band. However, the measured squeezing strength of −6.5 dB deviates from the ideal value predicted by Eq. (1). This is attributable to the fact that, aside from technical noise, optical losses and phase fluctuations related to squeezing preparation and propagation are inevitably introduced into the system. Optical losses introduce vacuum noise, while phase fluctuations project the anti-squeezed quadrature into a squeezed one. The combined effect reduces the observed squeezing strength (see Supplementary Note 4). The total optical loss and phase fluctuation^59^ are evaluated to be ${l}_{{\rm{t}}{\rm{o}}{\rm{t}}}=0.1$ and ${\theta }_{{\rm{t}}{\rm{o}}{\rm{t}}}=21$ mrad, respectively. Taking into account the total technical noise listed in Table 1, we theoretically simulate the squeezing strength based on Eq. (1), shown as trace (h). The experimental result is in good agreement with the theoretical prediction. Notably, the bandwidth of the bright squeezed light is identical to that of the squeezed vacuum, which is limited by the linewidth of the OPO. Here, we only show quantum noise within the frequency range up to 1 MHz.

**Table 1:** Budget of technical noise normalized to shot noise in passive and hybrid stabilizations

Source of technical noise (@50 kHz)	Passive (dB)	Passive and active (dB)
Amplitude noise	−11	−10
Electronic noise	−21	−14
Residual in-loop noise		−30
Total noise	−10.6	−8.5

**Fig. 5:**
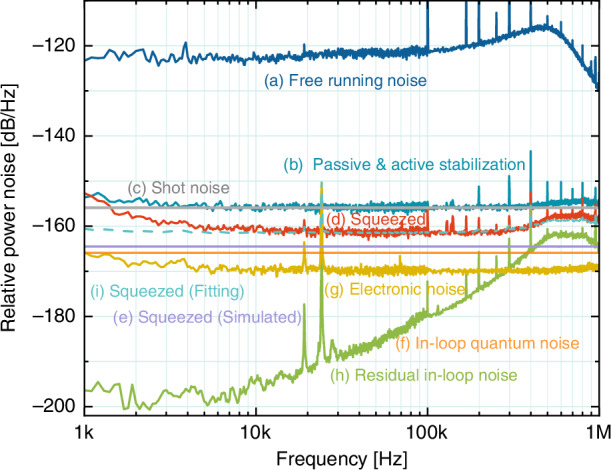
Measurement results of bright squeezed light at 1 mW with nonclassical hybrid passive–active stabilizations. Trace (a): measured technical noise of the free-running laser. Trace (b): measured RSN of the 1 mW optical power detected by ool-PD. Trace (c): theoretical shot noise at 1 mW. Trace (d): measured noise variance of the bright squeezed light. Trace (e): simulated noise variance of the squeezed light. Trace (f): quantum noise coupled through the in-loop empty port of the VBS. Trace (g): sum of the electronic noise of the two photodetectors. Trace (h): residual technical noise of the in-loop under hybrid stabilizations. Trace (i): uncorrelated sum of the noise sources (e)–(h). All measurements were performed using a spectrum analyzer (R&S FSW) at two frequency bands, 1–100 kHz and 100 kHz–1 MHz, with a RBW of 100 Hz and a VBW of 10 Hz

Subsequently, by employing the nonclassical hybrid passive-active power stabilization, we increase the output power of the bright squeezed light to 1 mW. Figure 5 presents the measured technical and quantum noises across the 1 kHz–1 MHz band. Trace (a) shows the amplitude noise of the free-running laser at 1 mW. Trace (b) indicates that, after stabilization, the amplitude noise reaches the theoretically predicted SNL (trace (c), −156 dB/Hz at 1 mW). The residual technical noise of the system is reduced to the level of trace (h), which is more than 24 dB below the out-of-loop beam’s SNL within 100 kHz. This noise level increases rapidly above 100 kHz due to reduced feedback gain at higher frequencies. Trace (f) represents the vacuum noise coupled from the empty port of the VBS in the optical path of the in-loop unit, which becomes the dominant factor to reduce the squeezing strength (see Supplementary Note 2). The electronic noise, shown in trace (g), is measured to be –170 dB/Hz. The total technical noise of the out-of-loop beam is the sum of traces (f)–(h), which remains below the SNL. As a result, a −5.5 dB bright amplitude squeezed light (trace (d)) at 1 mW is generated across 2 kHz–1 MHz band. Based on Eqs. (1)–(3), Eq. (S16) (see Supplementary Note 2) and the technical noises in Table 1, we numerically simulate the squeezing strength (trace (i)), which shows excellent agreement with the experimental results (trace (d)). The observed noise deterioration below kHz may be attributed to three key factors: (i) poor performance of the gain and electronic noise of our photodetectors in this frequency range; (ii) larger amplitude noise of the laser below kHz, inducing the SHG locking unstable; and (iii) noise transferring from the upstream MC.

The results in Fig. 4 indicate that the squeezed light generated by a single TSPR passive stabilization regime is primarily limited by the technical noise of the laser. Due to the intrinsic loss introduced by the BS and finite noise suppression capability, the passive interference technology with TSPR is suitable only for highly unbalanced BS configurations, enabling low-power-level squeezing generation (see Supplementary Note 3). Nevertheless, this regime can extend the squeezing bandwidth to the MHz frequency range. Further power scaling lowers the ${{\rm{R}}{\rm{S}}{\rm{N}}}_{{\rm{O}}{\rm{O}}{\rm{L}}}$ toward the technical noise floor of the laser, as shown in Fig. 1b, thereby narrowing the gap between the SNL and technical noise. This reduction diminishes the squeezing strength and can even completely destroy the squeezed state. In a nonclassical hybrid power stabilization regime, active feedback control compensates for the finite noise suppression capability of the passive approach and enhances the technical noise suppression gain. Consequently, the hybrid regime inherits both the maximum noise suppression ability of the active scheme and the broadband noise reduction capability of the passive one. This enables the preparation of bright squeezed light with higher power and stronger squeezing across kHz–MHz band, as shown in Fig. 5. Further improving in optical power, squeezing strength, and bandwidth are mainly constrained by the detectable power and electronic noise of the il-PD, as well as the bandwidth of the PID controller. The former sets the lower bound of achievable technical noise, while the latter determines the upper bound of the feedback bandwidth. Moreover, the Fourier frequency range of bright squeezing can be extended to hundreds of hertz or lower by introducing an additional active feedback control loop before the SHG to suppress technical noise in this range^60^.

## Discussion

We have demonstrated a novel bright amplitude squeezed light source by integrating a TSPR-based passive stabilization stage into a nonclassical active feedback scheme. The hybrid dual-stage noise stabilization strategy relaxes the requirements on the feedback loop gain while significantly extending the feedback bandwidth into the MHz range. We have also developed a comprehensive theoretical model for this regime, which can be used to determine the optimal parameters for nonclassical feedback stabilization. Finally, we have experimentally generated –5.5 dB bright squeezed light with output power of 1 mW and bandwidth of kHz–MHz range. To further improve the squeezing strength or power for the requirements of different application scenarios, the splitting ratio of the BS, as well as the balance between technical and shot noise, should be optimized based on the theoretical model. Our bright squeezed light source is well suited for a wide range of quantum metrology applications, such as biological tracking, cavity-mechanics strong coupling, quantum radiation pressure noise suppression, and quantum-enhanced displacement sensing, and so on.

## Materials and methods

### Low-phase-noise single-frequency fiber laser

A single-frequency CW laser (NKT X15) with a linewidth below 100 Hz is used as the laser source. The laser exhibits excellent low phase noise performance, which is beneficial to phase-sensitivity operation in bright squeezed light generation. It exhibits excellent phase noise characteristics, effectively mitigating noise coupling during relative phase-locking processes. As a result, it significantly reduces phase fluctuations and maintains the squeezing strength of the initial squeezed vacuum state with high fidelity.

### Passive noise stabilization with SHG

The SHG is a semi-monolithic cavity, which consists of a concave mirror driven by a piezoelectric transducer (PZT) and a periodically poled PPKTP crystal (dimensions: 10 mm × 2 mm × 1 mm). The convex surface of the crystal has a curvature radius of 12 mm, coated with high reflectivity (HR) for both 1550 and 775 nm wavelengths, while the planar surface is anti-reflection (AR) coated for both wavelengths. The concave mirror has a curvature radius of 30 mm, a transmissivity of 12 ± 1.5% at 1550 nm, and high transmission (HT) at 775 nm. The air gap between the two optical elements is 27 mm, giving a total cavity length of ~39 mm. The SHG exhibits a linewidth of 68 MHz and a finesse of 48. The mode-matching efficiency of the 1550 nm laser beam is optimized to ~99.6%. A resonant electro-optic phase modulator (EOPM) generates a pair of ±36 MHz sidebands for the PDH technique to stabilize the SHG cavity length. The phase-matching condition is achieved by precisely stabilizing the PPKTP crystal temperature at 36.0 °C (±0.1 °C) using a high-resolution temperature controller.

During the experiment, a 1550 nm laser beam at 500 mW is injected into the SHG to up-convert a 775 nm laser, which serves as the pump source for the OPO. Furthermore, the SHG process inherently provides passive power stabilization, effectively suppressing amplitude noise across the kHz–MHz frequency range. This stabilization mechanism arises from the nonlinear frequency conversion process, which establishes a nonlinear dependence between the intracavity circulating power and the reflected fundamental wave. A maximum amplitude noise reduction of 35 dB is achieved as the slope of this nonlinear transfer function approaches zero, corresponding to a conversion efficiency of 70%. At this point, the noise is reduced to near the SNL among the kHz–MHz frequency range^52^. Then, the passively stabilized 1550 nm beam, with an output power of 110 mW, is directly delivered to the downstream experiments.

### Active noise stabilization with a high-gain broadband feedback loop

We employ a broadband PID controller (Vescent, Model D2-125, 2 MHz bandwidth) in combination with a gain-adjustable photodetector (Newport, Model 2053, 10 MHz bandwidth) to construct an 80 dB flat gain feedback control in the kHz frequency range. The photodiode of the detector is replaced with a high quantum efficiency (>99%) one to minimize the optical loss in squeezed state detection. It has a 100 μm diameter of active area, and is AR-coated at 1550 nm at 20° for s-polarization. The detector also has a low electronic noise of −170 dB/Hz. An electro-optic amplitude modulator (EOAM) (Thorlabs: EO-AM-NR-C3) acts as the actuator for amplitude noise suppression. The amplitude noise of its output is sensed by the il-PD positioned at the reflected port of the 99:1 BS. To avoid excess noise coupling, no external amplifier is used to drive the EOAM. With these optimizations, a high-gain feedback control loop is realized with 1 kHz–1 MHz frequency bandwidth.

## Supplementary information

Supplementary Information

## Data Availability

The authors declare that all data supporting the findings of this study can be found within the paper. Additional data supporting the findings of this study are available from the corresponding authors (Y.J.W. and Y.H.Z.) upon reasonable request.
