# Supplementary material for: Bright squeezed light in the kilohertz frequency band
Source: Light Sci Appl. 2025 Sep 8;14:310. doi: 10.1038/s41377-025-02013-7 (PMC12420786; doi:10.1038/s41377-025-02013-7)
Supplement: Supplementary file 1 — Supplementary Information [file 41377_2025_2013_MOESM1_ESM.pdf]

Supplementary Information for:

Bright squeezed light in the kilohertz frequency band

Ruixin Li[1], Bingnan An[1], Nanjing Jiao[1], Junyang Liu[1], Lirong Chen[1,2], Yajun Wang[1,2,*], and Yaohui Zheng[1,2,**]

[1]*State Key Laboratory of Quantum Optics Technologies and Devices, Institute of Opto-Electronics, Shanxi University, Taiyuan 030006, China*

[2]*Collaborative Innovation Center of Extreme Optics, Shanxi University, Taiyuan 030006, China.*

[*]*Corresponding author: YJWangsxu@sxu.edu.cn*

[**]*Corresponding author: yhzheng@sxu.edu.cn*

This document provides supplementary materials for the manuscript titled "Bright squeezed light in the kilohertz frequency band". It is organized into four sections: Supplementary Note 1. Theoretical analysis of bright squeezed light generation via passive interference; Supplementary Note 2. Theoretical analysis of bright squeezed light generation via nonclassical active feedback; Supplementary Note 3. Comparation between the passive interference and active feedback regimes; Supplementary Note 4. Loss and phase fluctuation budget for hybrid nonclassical stabilization.

Supplementary Note 1. Theoretical analysis of bright squeezed light generation via passive interference

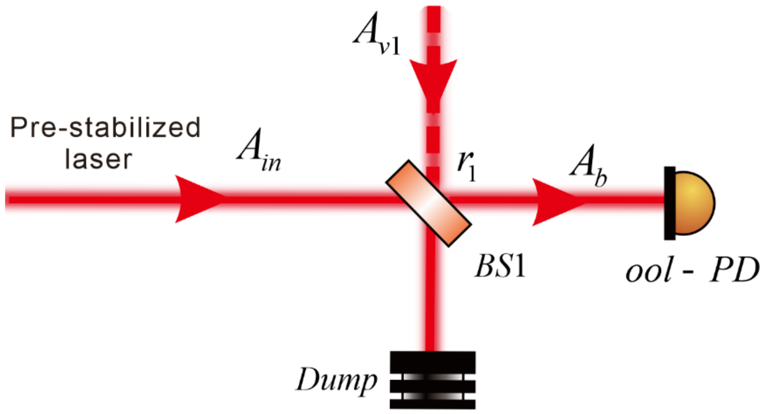

Fig. S1 Schematic of bright squeezed light generation via passive interference. Diagram illustrating how a pre-stabilized laser interferes with squeezed vacuum at a beam splitter (BS1) to produce bright amplitude-squeezed light, which supports the derivation of Eqs. (S1)–(S6).

Figure S1 illustrates the bright squeezed light generation diagram via passive interference. A pre-stabilized laser[1,2] A_{in} interferes with a squeezed vacuum at a beam splitter (BS1). The amplitude quadrature fluctuation of the output field is given by:

$$\delta A_b = -\sqrt{r_1}\delta A_{v1} + \sqrt{1-r_1}\delta A_{in} \qquad (S1)$$

here, r_1 is the power reflectivity of BS1, δA_{in} is the amplitude noise of the input field, and δA_{v1} represents the fluctuation of squeezed vacuum.

The noise variance of output field δA_b becomes:

$$V_b = r_1 V_{v1} + (1-r_1)V_{in} \qquad (S2)$$

The input noise V_{in} comprises both shot noise and technical noise, i.e., $V_{in} = V_{sn} + V_{tn}$. Substituting this into Eq. (S2) yields:

$$V_b = r_1 V_{v1} + (1-r_1)(V_{sn} + V_{tn}) \qquad (S3)$$

Multiplying both sides of Eq. (S3) by the relative shot noise of output field[3], i.e., $RSN_{OOL} = 2h\nu/P_{OOL}$, where h is Planck's constant, ν is the laser frequency, and P_{OOL} is the optical power of the output field, we obtain:

$$RSN_{OOL}V_b = RSN_{OOL}r_1 V_{v1} + (1-r_1)RSN_{OOL} + g(f)\cdot TN_F \qquad (S4)$$

where $g(f) = V_{output}/V_{input}$ represents the passive noise suppression factor introduced by the three-step photon recycling (TSPR) stabilization scheme[1], where V_{input} and V_{output} are the noise variances before and after stabilization, respectively.

TN_F denotes the relative technical noise of the free-running laser.

Rearranging Eq. (S4) gives the total noise variance of bright squeezed field at the output port of BS1:

$$V_b = r_1 V_{v1} + (1 - r_1) + \frac{TN_{OOL-P}}{RSN_{OOL}} \quad (S5)$$

where $TN_{OOL-P} = g(f) \cdot TN_F$ represents the residual technical noise after passive stabilization. When the power reflectivity of the BS1 approaches to unity ($r_1 \approx 1$), the output noise variance simplifies to:

$$V_b \cong V_{v1} + \frac{TN_{OOL-P}}{RSN_{OOL}} \quad (S6)$$

Supplementary Note 2. Theoretical analysis of bright squeezed light generation via nonclassical active feedback

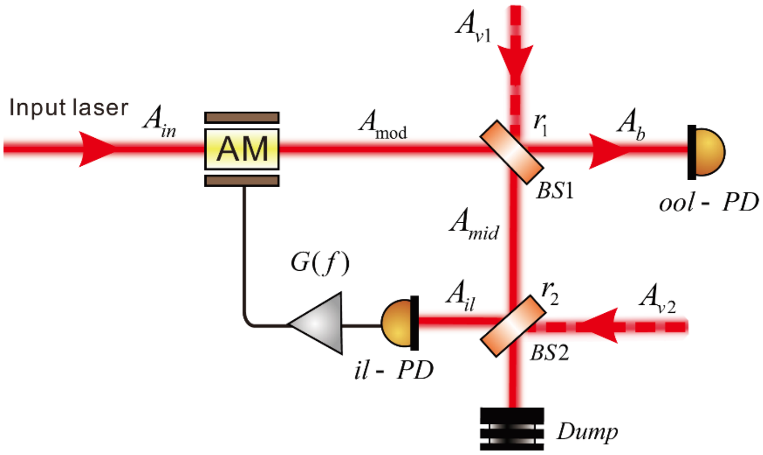

Fig. S2 Schematic diagram of bright squeezed light generation via nonclassical active feedback. The figure provides a detailed representation of the feedback loop, including squeezed vacuum injection via BS1, power attenuation via BS2, and closed-loop feedback. This model forms the basis for deriving Eqs. (S7)–(S17).

Figure S2 illustrates the nonclassical active feedback scheme for theoretical analysis. The input laser beam A_{in} enters into a nonclassical active feedback control loop, where it interferes with a squeezed vacuum at the first BS1. After this interfering, the beam is attenuated by a second BS2, and subsequently detected by an in-loop photodetector (il-PD). The output current from the il-PD is fed back to an upstream amplitude modulator (AM), enabling active suppression of amplitude quadrature noise. The relation between the involved optical fields can be described by[4-6]:

$$\delta A_{mod} = \delta A_{in} + G(f)\delta A_{ilPD} \quad (S7)$$

$$\delta A_{mid} = \sqrt{r_1}\delta A_{mod} + \sqrt{1-r_1}\delta A_{v1} \qquad (S8)$$

$$\delta A_{il} = \sqrt{r_2}\delta A_{mid} + \sqrt{1-r_2}\delta A_{v2} \qquad (S9)$$

$$\delta A_{ilPD} = \delta A_{il} + \delta e_{ilPD} \qquad (S10)$$

$$\delta A_b = \sqrt{1-r_1}\delta A_{mod} - \sqrt{r_1}\delta A_{v1} \qquad (S11)$$

here, $G(f)$ is the frequency-dependent gain of the nonclassical active feedback system, which represents the noise suppression capability in the feedback bandwidth; δA_{ilPD} and δA_{mod} are the amplitude noises readout by il-PD and exporting from the AM, respectively; r_1 and r_2 are the power reflectivities of BS1 and BS2, respectively; δA_{v1} and δA_{v2} denote the squeezed vacuum and vacuum noise entering through the unused ports of the two BSs, respectively; δA_{mid} and δA_{il} are the amplitude noises of the optical field between BS1 and BS2 and entering the il-PD, respectively; δe_{ilPD} is the electronic noise of the il-PD.

Substituting Eqs. (S7)–(S10) into Eq. (S11), we obtain the fluctuation of output field δA_b as:

$$\delta A_b \cong \frac{\sqrt{1-r_1}\delta A_{in} + \delta A_{v1} G(f)\sqrt{r_2}}{(1-G(f)\sqrt{r_1 r_2})} +$$

$$\frac{G(f)\sqrt{1-r_1}\sqrt{1-r_2}\delta A_{v2} + \sqrt{1-r_1}G(f)\delta e_{ilPD}}{(1-G(f)\sqrt{r_1 r_2})} \qquad (S12)$$

Under the condition of high feedback gain, $G(f) \gg 1$, the noise variance of the output field becomes:

$$V_b \cong \frac{(1-r_1)V_{in}}{(1-G(f)\sqrt{r_1 r_2})^2} + \frac{V_{v1}}{r_1} + \frac{(1-r_1)(1-r_2)V_{v2}}{r_1 r_2} + \frac{(1-r_1)V_e}{r_1 r_2} \qquad (S13)$$

Multiplying both sides of Eq. (S13) by the relative shot noise of the output field, RSN_{OOL}, yields the relative noise form as[3]:

$$RSN_{OOL}V_b = \frac{RSN_{in}V_{in}}{(1-G(f)\sqrt{r_1 r_2})^2} + RSN_{OOL}\frac{V_{v1}}{r_1} + RSN_{IL}(1-r_2)V_{v2} + RSN_{IL}V_e \qquad (S14)$$

here, RSN_{in} and RSN_{IL} are the relative shot noises of the laser beams of the input and in-loop, respectively. The noise of the input field includes the shot and technical noises, $V_{in} = V_{sn} + V_{tn}$, and can be expressed as $RSN_{in}V_{in} = RSN_{in} + g(f) \cdot TN_F$. Accordingly, the noise variance of the output bright squeezed light becomes:

$$V_b = \frac{V_{v1}}{r_1} + \frac{\frac{RSN_{in} + g(f) \cdot TN_F}{(1 - G(f)\sqrt{r_1 r_2})^2} + RSN_{IL}(1 - r_2)V_{v2} + RSN_{IL}V_e}{RSN_{OOL}} \qquad (S15)$$

In our scheme, the input laser is first passively pre-stabilized before applying nonclassical active feedback. In general, $V_{tn} \gg V_{sn}$, and the technical noise of the output field after hybrid passive and active stabilization can be defined as:

$$TN_{OOL-P\&A} \cong \frac{g(f) \cdot TN_F}{(1 - G(f)\sqrt{r_1 r_2})^2} + RSN_{IL}(1 - r_2)V_{v2} + RSN_{IL}V_e \qquad (S16)$$

The first term in Eq. (S16) represents the residual technical noise of the out-of-loop unit, which in principle can be fully eliminated by an infinite feedback gain $G(f)$. The second term reflects the quantum noise introduced by the vacuum field coupled from BS2, and its magnitude depends on the reflectivity r_2 and the in-loop shot noise limit. The last term represents the electronic noise of the feedback control loop.

When the entire optical power is detected by the il-PD, i.e., $r_2 \approx 1$. Eq. (S16) simplifies to Eq. (3) in the main text. Under nonclassical active stabilization, the noise variance of the output bright squeezed light can be expressed as:

$$V_b = \frac{V_{v1}}{r_1} + \frac{TN_{OOL-P\&A}}{RSN_{OOL}} \qquad (S17)$$

Supplementary Note 3. Comparation between the passive interference and active feedback regimes

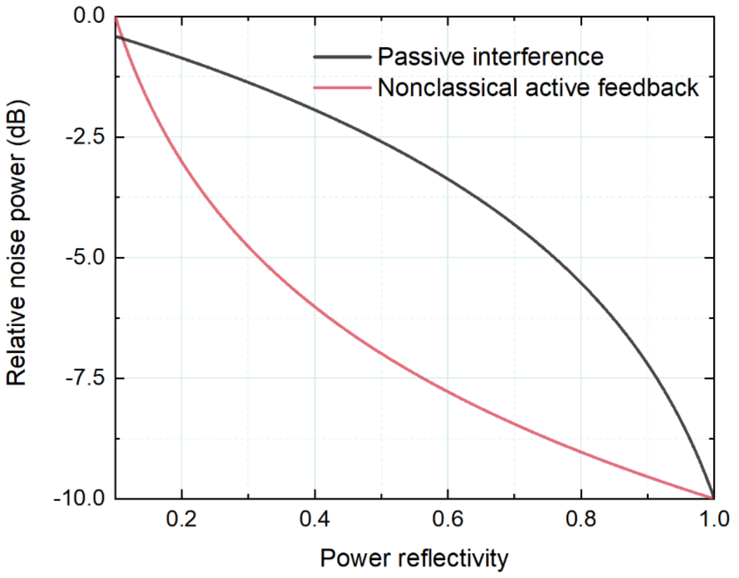

Fig. S3 Noise power at the output of BS1 for passive interference versus nonclassical active feedback schemes with different power reflectivity of BS1.

TABLE. S1 Examples of passive interference and nonclassical active feedback regimes for bright

squeezing generation at different splitting ratio (r_1: t_1) of BS1.

BS1 ratio (r_1: t_1)	Squeezing strength (dB)		Squeezing power (mW)
	Passive interference	Nonclassical active feedback	
99:1	-9.63	-9.96	1
90:10	-7.21	-9.54	10
50:50	-2.60	-6.99	50
20:80	-0.87	-3.01	80
10:90	-0.41	0	90

We compare the performance of passive interference and nonclassical active feedback schemes for generating bright squeezed light as a function of the power reflectivity r_1 of BS1. In this analysis, we assume that the technical noise of a 100 mW input laser beam is completely eliminated, and a -10 dB squeezed vacuum is injected at BS1 (see Figs. S1 and S2). Based on Eqs. (S5) and (S17), the squeezing strengths of the two schemes are calculated as shown in Fig. S3. Table S1 summarizes the squeezing strength and power levels at different splitting ratio (r_1: t_1) of BS1.

Apparently, as the power reflectivity decreases, more optical power can be extracted to scale the power of the bright squeezed light. However, squeezing strength is reduced gradually due to increased vacuum noise coupling. From Fig. S3 and Table S1, we can infer that the nonclassical feedback scheme always owns stronger squeezing strength than passive interference one at different splitting ratio, except $r_1 < 0.11$. Even with a highly imbalanced BS, e.g., 99% reflectivity, the nonclassical active feedback regime still presents a 0.33 dB noise suppression enhancement than the passive interference one. We can enlarge the difference between the two regimes by changing the splitting ratio to a balanced BS, which is also benefited to increase the power level of the nonclassical light. Therefore, to achieve an optimal balance between squeezing strength and output power for different application scenario, the BS' splitting ratio must be carefully optimized.

Supplementary Note 4. Loss and phase fluctuation budget for hybrid nonclassical stabilization

TABLE. S2 Budget of the optical loss and phase fluctuation relating to the squeezing preparation

**Source of optical loss**	Relevant loss (%)
OPO escape efficiency	3.0±0.3
Efficiency of interference	2.8±0.2
Quantum efficiency of photodiodes	1.0±0.2
Propagation efficiency	3.2±0.5
Total efficiency	10±0.8
**Source of phase fluctuation**	Value (mrad)
OPO	2±0.1
Relative phase between squeezed and frequency-shifted light	8±0.2
Relative phase of squeezed and local field	11±0.5
Total phase fluctuation	21±0.8

Table S2 summarizes the optical loss and phase fluctuation budget in our experimental setup. The total optical loss, including the escape efficiency of the optical parametric oscillator (OPO), interference visibility, quantum efficiency of photodiode and propagation loss, is approximately 10%, which introduces vacuum noise to the squeezing quadrature, and limits the achievable squeezing level. While the total phase fluctuation is approximately 21 mrad, which degrades the measured squeezing by mixing the anti-squeezing quadrature into the squeezing one.

By considering the total optical loss l_{tot} and phase fluctuation θ_{tot}, the variances $V_{a/s}$ of anti-squeezing and squeezing quadratures can be expressed as[7,8]

$$V_{a/s} = \left[1 \pm \frac{4(1-l_{tot})\sqrt{p/p_{th}}}{\left(1 \mp \sqrt{p/p_{th}}\right)^2 + 4(f/k)^2}\right] cos^2\,\theta_{tot} + \left[1 \mp \frac{4(1-l_{tot})\sqrt{p/p_{th}}}{\left(1 \pm \sqrt{p/p_{th}}\right)^2 + 4(f/k)^2}\right] sin^2\,\theta_{tot} \qquad (S18)$$

where p, and p_{th} are the pump and threshold powers of the OPO, respectively; f and k denote the Fourier frequency and cavity linewidth of the OPO, respectively. Based on Eq. (S18), $l_{tot} = 10\%$, $\theta_{tot} = 21$ mrad, $p = 10$ mW, $p_{th} = 20$ mW and $k = 110.4$ MHz, the observed squeezing strength of -8.6 dB agrees well with the theoretical value.

Reference

1. Jiao, N. et al. Passive laser power stabilization in a broadband noise spectrum via a second-harmonic generator. *Opt. Lett.* **49**, 3568-3571 (2024).
2. Yap, M. J. et al. Broadband reduction of quantum radiation pressure noise via squeezed light injection. *Nat. Photonics* **14**, 19–23 (2020).
3. Vahlbruch, H., Wilken, D., Mehmet, M. & Willke, B. Laser power stabilization beyond the shot noise limit using squeezed light. *Phys. Rev. Lett.* **121**, 173601 (2018).
4. Lam, P. K., Ralph, T. C., Huntington, E. H. & Bachor, H. A. Noiseless signal amplification using positive electro-optic feedforward. *Phys. Rev. Lett.* **79**, 1471 (1997).
5. Buchler, B. C. et al. Feedback control of laser intensity noise, *Phys. Rev. A* **57**, 1286 (1998).
6. Zhang, J., Ma, H., Xie, C. & Peng, K. Suppression of intensity noise of a laser-diode-pumped single-frequency Nd:YVO_4 laser by optoelectronic control. *Appl. Opt.* **42**, 1068 (2003).
7. Dwyer, S. et al. Squeezed quadrature fluctuations in a gravitational wave detector using squeezed light. *Opt. Express* **21**, 19047-19060 (2013).
8. Yang, W. et al. Detection of stably bright squeezed light with the quantum noise reduction of 12.6 dB by mutually compensating the phase fluctuations. *Opt. Lett.* **42**, 4553-4556 (2017).
